# Cerebellar Ataxia in the Setting of Hashimoto’s Thyroiditis: A Case Report Update and Review

**DOI:** 10.7759/cureus.45959

**Published:** 2023-09-25

**Authors:** Gustavo Chiriboga Reyes, Elisa Pallares Vela, Peter G Bernad

**Affiliations:** 1 Neurology, Neurology Services, Inc, Washington, USA; 2 Neurology, George Washington University Hospital, Washington, USA

**Keywords:** hashimoto’s thyroiditis, cerebellar-ataxia, autoimmune cerebellar ataxia, anti-thyroid peroxidase antibodies, thyroid peroxidase antibody, hashimoto’s encephalopathy

## Abstract

Hashimoto's encephalopathy (HE) is a rare diagnosis. Establishing the diagnosis itself is quite challenging, as symptoms vary among cases and there is still no standard confirmatory test. The clinical presentation is heterogeneous; however, patients with HE most commonly experience focal neurological deficits, frequently accompanied by cognitive dysfunction, aphasia, or paresis. The most widely recommended initial treatment for cases of HE is a course of corticosteroids. Nonetheless, their response varies from patient to patient, and some may become resistant to them. There are many proposed second-line treatments; however, there is little data and no consensus on the best alternative treatment when steroid therapy fails. This article provides an update on a case of cerebellar ataxia in a 30-year-old female patient with Hashimoto’s thyroiditis. She initially presented with rapid-onset progressive symptoms of cerebellar ataxia (movement incoordination, dysmetria, and balance problems) and had elevated serum anti-thyroid peroxidase antibodies. She was diagnosed with HE and was initially treated with methylprednisolone. However, her symptoms recurred after tapering steroid therapy, and eventually, they ceased to manage her symptoms, plus she developed steroid-induced osteoporosis. She began treatment with intravenous immunoglobulin (IVIG) as an alternative in April 2022. Since then, she has had four infusions of IVIG that have allowed her to remain symptom-free.

## Introduction

Hashimoto’s encephalopathy (HE) is a rare, non-infectious encephalitis, associated with Hashimoto’s thyroiditis [1,2]. It has an estimated prevalence of 2.1 in 100,000 subjects, with a higher predisposition in female patients [1,3]. Due to its response to corticosteroid treatment, it is also known as steroid-responsive encephalopathy associated with autoimmune thyroiditis (SREAT) [1]. Even when it is considered an inflammatory autoimmune disorder, its precise etiology and pathogenesis remain unclear [4].

HE is a syndrome with a variety of clinical presentations; neurologic and psychiatric manifestations are common, but cerebellar ataxia is unusual, except in children [4,5]. The diagnosis is clinical, and while it may be associated with increased levels of antithyroid antibodies, the majority of affected patients are euthyroid or slightly hypothyroid at the time of diagnosis [1,4]. At this time, HE continues to be a diagnosis of exclusion as no specific biomarkers for the disease have been identified [6,7]. The initial treatment is corticosteroids, but in corticosteroid-resistant HE, or when there are relapses after treatment, other pharmacological approaches should be considered [6,8]. In resistant cases, combinations of corticosteroids with azathioprine, methotrexate, rituximab, or cyclophosphamide have been recommended [1,8,9]. Plasmapheresis and intravenous immunoglobulin (IVIG) have also proved to be effective in selected patients [1,8].

## Case presentation

Our colleagues previously published this case of a 30-year-old woman diagnosed with HE [10]. In July 2019, the patient initially presented with acute onset tremors and loss of dexterity in both hands. Afterward, she developed slurred speech and weakness of all extremities. Eventually, she could not walk by herself, and her speech became unintelligible. The gravity of her symptoms prompted her to go to the local emergency department (approximately three hours after the initial onset of symptoms), where they initially did a complete blood count, routine metabolic panel, routine urinalysis test, CT, and MRI. The results revealed no abnormalities. Lumbar puncture and CSF analysis were also done at the ER visit and ruled out Lyme disease and West Nile virus encephalitis. After ruling out an acute cause for the patient’s symptoms, she was discharged from the emergency department and referred to a neurologist.

Two weeks later, in August, she continued to experience the previously mentioned symptoms. Additionally, she experienced new cerebellar symptoms (dysmetria, bradykinesia, movement incoordination, and loss of balance) and emotional lability demonstrating pseudobulbar symptoms. She denied having diplopia, vertigo, hearing problems, psychiatric symptoms, memory deficit, lack of concentration, or cognitive difficulties. Prior to her illness, the patient was active and healthy overall, except for subclinical hypothyroidism, which was adequately controlled with 50 mcg of levothyroxine once a day. Her family history was positive for Grave’s disease.

On initial physical examination in the neurology service (in September 2019), the patient had intention tremor, dysmetria, dysdiadochokinesia, abnormal tandem gait, and postural imbalance upon standing. No other neurologic deficits were noted at that moment. 

The initial and subsequent results of tests conducted during follow-up are summarized in Table 1. Her clinical presentation, the biopsy finding of Hashimoto’s thyroiditis, the increased levels of thyroid peroxidase (TPO) antibodies and the exclusion of other diagnoses supported our belief that the patient had HE with cerebellar ataxia.

**Table 1:** Summary of laboratory test results over three years of follow-up. EEG: electroencephalogram, IV: intravenous, MRI: magnetic resonance imaging, MMSE: mini mental status exam. *Laboratory method used for TPO antibodies: Immunoassay for quantitative determination of TPO antibodies.

Date	Test	Result
July 2019	CT of the head without IV contrast	No acute intracranial hemorrhage, midline shift, or mass effect, and no CT evidence of acute intracranial abnormality
August 2019	MRI of the head without IV contrast	No acute intracranial abnormality. No infarct.
	Blood serum and CSF analysis	Ruled out Lyme disease and West Nile virus infection, normal cell count, normal urinalysis. Normal metabolic panel. Negative toxic panel.
September 2019	Ataxia, common repeat expansion evaluation	Ruled out genetic variants associated with hereditary ataxia. Serum evaluation ruled out other autoimmune disorders.
January 2020	Thyroid peroxidase (TPO) antibody*. Normal range: 0–34 IU/mL	Elevated (158 IU/mL)
	Thyroxine (T4). Normal range: 4.5–12.0 ug/dL	Normal (6.7 ug/dL)
February 2020	Head MRI with and without contrast	Unremarkable MRI examination of the brain
March 2020	Thyroid peroxidase (TPO) antibody*. Normal range: 0–34 IU/mL	Elevated (127 IU/mL)
	Thyroid-stimulating hormone (TSH). Normal range: 0.27–4.2 uIU/mL	Normal (2.72 uIU/mL)
	Thyroxine (T4). Normal range: 4.5-12.0 ug/dL	Normal (6.4 ug/dL)
	Triiodothyronine (T3). Normal range: 80-200 ng/dL	Normal (91.1 ng/dL)
	Thyroglobulin (TG) antibody. Normal range: 0.0-0.9 IU/mL	Normal (<0.9 IU/mL)
September 2020	Thyroid biopsy	Hashimoto's thyroiditis
June 2021	Thyroid peroxidase (TPO) antibody*. Normal range: 0–34 IU/mL	Elevated (306 IU/mL)
July 2021	MMSE	Normal
	EEG	No epileptiform discharges
October 2021	Thyroid peroxidase (TPO) antibody*. Normal range: 0–34 IU/mL	Elevated (249 IU/mL)
	Thyroid-stimulating hormone (TSH). Normal range: 0.27–4.2 uIU/mL	Normal (2.78 uIU/mL)
	Free thyroxine (T4). Normal range: 0.93-1.7 ng/dL	Normal (1.32 ng/dL)
	Triiodothyronine (T3). Normal range: 80-200 ng/dL	Normal (87.0 ng/dl)
	Thyroglobulin (TG) antibody. Normal range: 0.0-0.9 IU/mL	Normal (<0.9 IU/mL)
February 2022	Thyroid peroxidase (TPO) antibody*. Normal range: 0–34 IU/mL	Elevated (254 IU/mL)
	Thyroid-stimulating hormone (TSH). Normal range: 0.27–4.2 uIU/mL	Normal (1.88 uIU/mL)
	Triiodothyronine (T3). Normal range: 80-200 ng/dL	Normal (91.9 ng/dl)
	Thyroglobulin (TG) antibody. Normal range: 0.0-0.9 IU/mL	Normal (<0.9 IU/mL)
March 2022	MRI of the head without IV contrast	Stable punctate region of susceptibility in the posterior right lateral aspect of the pons compared with a brain MRI study dated February 2020

Her initial treatment approach included a two-week course of corticosteroids. After the first course, she became symptom-free for two weeks. After a few days of steroid treatment, the patient regained her sense of balance; she had more control over her hands and managed to handle things like a mug without spilling or for the mug to accidentally slip from her hands; and her ability to articulate words improved as well. The later courses all had progressively shorter symptom-free periods until the last one had no symptom-free period after stopping the medication. Within a year, she was treated with six two-week courses of methylprednisolone (from 2019 to 2022). Her pseudobulbar symptoms were controlled with a combination of dextromethorphan and quinidine. Some of her symptoms never completely resolved with the use of steroids, like her ability to do tasks that required fine hand movements or her ability to do very physically demanding tasks. Additionally, she developed osteopenia of the hip, likely due to her prolonged corticosteroid treatment.

Patient progress after starting IVIG treatment

Complete remission of symptoms was not achieved despite the three courses of steroids. After the steroid courses, a new TPO measurement was done; it was higher than her previous measure. Levothyroxine was increased from 50 mcg to 75 mcg. Due to her lack of response to steroid therapy, a second-line treatment was considered, and she had her first IVIG course from April 25th to April 29th, 2022. After her first course, she was able to ride horses and do other physically demanding activities; her speech became clearer compared to the previous improvements with the steroid courses; and she could finally do fine motor hand movements with ease, especially writing. Her pseudobulbar symptoms also improved after IVIG therapy.

During the scheduled IVIG sessions, the patient initially received 10% IVIG Gamma KED 25 grams over a period of four days, administered via an IV infusion pump. The maximum infusion rate is 261 ml/h, but it is modified to the patient's tolerance. The patient received two medications 30 minutes prior to the infusion: acetaminophen 650 mg PO and diphenhydramine 25 mg PO. One additional IV drip was connected with normal saline to infuse IVIG treatment if needed. The patient is currently taking 50 grams of 10% IVIG Gamma KED administered following the same protocol as before. The patient has already had four IVIG infusion sessions. Usually, she experiences slight tremors approximately three to four weeks after her sessions, but these symptoms improve after the fourth day of IVIG. She has also experienced mild and episodic headaches throughout the month; however, they are bothersome. 

## Discussion

HE definition, incidence, and prevalence

HE is a noninfectious encephalitis of probable autoimmune origin that needs to be considered as a diagnosis of exclusion in patients who present with a wide range of neurological and psychiatric symptoms accompanied by normal or nonspecific MRI and CSF findings, and increased serum levels of thyroid peroxidase antibodies (TPO Ab) [2,6]. HE presentation can vary, and it usually has a relapsing and remitting course [2,5]. Our patient presented cerebellar symptoms at onset (dysmetria, movement incoordination, and loss of balance). Prospective studies of patients presenting with unexplained encephalopathy and detected antithyroid antibodies have estimated a prevalence of 2.1 in 100,000 subjects, with a female-to-male ratio of 4:1 [1,3]. Women in their 40s and 60s are more affected, but more severe cases have been described in males [4,11]. Although it is more frequent in adults, cases have been reported in both children and adults worldwide [11]. One-third of the patients with HE has been observed to have comorbid rheumatological and autoimmune diseases [2-4].

Pathogenesis

Impaired brain functioning is a hallmark of HE [3]. HE most frequently presents with subacute cognitive dysfunction (36-100%), altered consciousness (26-85%), stroke-like symptoms (18-31%), tremor (28-84%), myoclonus (37-65%), seizures (52-66%), and hallucinations [2,4,6]. Less common presentations include gait disorder or cerebellar ataxia (28-65%), status epilepticus (12-20%), and psychosis [4,6]. There are three major proposed pathophysiological causes of HE: (a) cerebral vasculitis, (b) autoantibodies, and (c) hormone dysregulation or toxicity [2,4]. It also seems that some HE cases may actually belong to the family of autoimmune encephalitis [4]. Despite many efforts to unearth the pathophysiology of this disease, it still remains unclear [4,5].

Diagnostic criteria

The diagnosis of HE is quite challenging since there is no gold/standard test for its diagnosis [3]. It should be suspected in any case of autoimmune thyroiditis with obvious neuro/psychiatric symptoms, regardless of the thyroid function status, since it is oftentimes normal at presentation [4,6]. Antithyroid antibodies are a hallmark of this disease; nonetheless, they are non-specific since they can be present in up to 13% of healthy individuals, 27% in healthy white women in their 60s, and patients with other autoimmune encephalitis [2,8,9,11]. Anti-TPO levels do not correlate with the severity of the disease, but some authors have considered them to be a good marker for treatment response [2,8]. Antibodies against a-enolase (amino NH2/terminal domain) have been identified in up to 68% of cases with HE; nonetheless, these have also been found in patients with other autoimmune disorders and in a few healthy people [6,8]. Graus and colleagues have proposed a clinical approach to its diagnosis that includes six diagnostic criteria (the patient must meet all six to be diagnosed as HE, according to Graus and colleagues), all of them were fulfilled by our patient [9] (Table 2).

**Table 2:** Diagnostic criteria for Hashimoto’s encephalitis. Adaptation from [8].

	Criteria	Our patient
1	Encephalopathy with seizures, myoclonus, hallucinations, or stroke-like episodes	Yes
2	Subclinical or mild over thyroid disease	Yes
3	MRI of the brain: normal or non-specific abnormalities	Yes
4	Serum thyroid antibodies for thyroid peroxidase or thyroglobulin	Yes
5	Absence of well-characterized neuronal antibodies in serum and CSF	Yes
6	Exclusion of alternative causes	Yes

Cerebellar ataxia

Gait disorder and cerebellar ataxia have been described by some authors with a prevalence of 28-65% [1,4]. Nonetheless, clinical reports of HE presenting with isolated cerebellar ataxia as the main symptom are rare [7]. Hashimoto’s encephalitis with cerebellar ataxia (HECA) has been shown to be more prevalent in patients of Asian origin than in patients of European origin in some studies [7]. As in HE, in HECA there is also a predominance of women over men [1,7]. There are various etiologies of cerebellar ataxia; some of them are stroke, infections, toxicities, paraneoplastic syndromes, structural diseases, metabolic causes, and vitamin deficiencies [7]. Other causes of cerebellar ataxia should be excluded in cases of suspected HECA.

Treatment

Most cases of HE (over 90%) have shown a good response to treatment with high doses of corticosteroids [1,3]. This is one of the reasons why they renamed it steroid-responsive encephalopathy associated with autoimmune thyroiditis (SREAT) [4,12]. Nonetheless, some studies have found that only 31% of patients with suspected HE showed a complete clinical response to steroid treatment [4]. There are currently no good predictors of treatment response, but close monitoring is important to identify resistance to corticoid treatment [4,6]. Our patient initially responded well to a course of corticosteroids; after three days of a two-week course, she was symptom-free. Short-course treatment can be effective, but relapses may also occur when treatment is shortly or abruptly stopped [3]. As in the case with our patient, after corticosteroids were tapered off, her symptoms relapsed. In resistant cases, some authors have recommended combinations of corticosteroids with azathioprine, methotrexate, rituximab, or cyclophosphamide [1,3,8]. Other options are plasmapheresis and intravenous immunoglobulin (IVIG) [1,3,13].

IVIG and our patient

Our patient did respond initially to corticosteroid medication. It is important to note that our patient had elevated levels of anti-thyroid peroxidase; however, these did not exceed 1000. In some cases, having higher levels of anti-TPO (more than 1000) was suggested to be related to a poor response to steroid therapy [6]. This correlates in a way with our patient because her initial anti-TPO levels were 158 in 2020; at the time, steroids worked well for her. However, steroids progressively stopped working, and the serum levels were almost 100 points higher in 2021-2022, previous to the start of IVIG therapy. Therefore, there might be a relationship between the amount of anti-thyroid peroxidase antibodies and the feasibility of treating a patient only with corticosteroids [6]. However, others have disputed the idea that pretreatment features (including anti-TPO levels) are related to steroid treatment response [4]. 

In a case report, IVIG improved the neurological outcomes in about 85-90% of autoimmune encephalitis in their first week [14]. By 29 days, there is a slight decrease in the patient population without symptoms (up to 80% at day 29 are symptom-free) [14]. There are no clinical practice guidelines for HE. We chose IVIG, in consensus with our patient, because it decreases immune cell activation (leading to less inflammation in the affected area) and our own firsthand experience with IVIG and its successful utilization in a multitude of other patients [14]. Our patient on her ongoing therapy with IVIG had very mild recurring symptoms every four weeks; she experienced tremors and a slight balance disturbance. 

The literature and our patient’s case support the evidence that IVIG is a safe treatment for patients with Hashimoto’s encephalitis [14]. The most common adverse events are transfusion reactions, for example, mild skin eruption, shivers, mild chest pain [4,14], and, as in the case of our patient, a headache. It is important to mention that there can be more important but rare complications, such as aseptic meningitis, anaphylaxis, acute renal failure, or intracerebral hemorrhage [4,14]. However, these types of events tend to be fairly uncommon [4,14].

## Conclusions

The presence of anti-thyroid antibodies, neurologic symptoms, and the exclusion of other differential diagnoses supports the diagnosis in favor of HE in our patient. Despite having serum anti-thyroid levels lower than 1000, corticosteroid response decreased over time in our patient; hence the correlation with pretreatment features and the probability of success of symptom remission with corticosteroid treatment remains unclear. More studies should be targeted toward elucidating the relationship between these two features, as they could greatly assist in refining therapies with enhanced precision and efficacy.

Having scarce data on IVIG treatment for HE made the decision to switch the initial treatment plan a very challenging one. That is why we highlight our case as an example of the positive impact that IVIG may have on the neurological symptoms and the quality of life of a patient with HE with previous corticosteroid response failure. IVIG treatment improved symptom-free time in our patient; it may also improve it in other patients with HE who have shown poor response to other treatments. In our case, IVIG treatment was a safe option; however, it is important to be aware of the rare but severe complications this treatment might cause. More comprehensive studies should be done to elucidate a more accurate percentage of severe complications and to better ascertain the safety profile of IVIG treatment in the setting of HE. More studies should be done to evaluate the long-term side effects of IVIG treatment for HE.
